# Assessment of treatment outcomes in patients receiving high- versus low-dose sulfamethoxazole-trimethoprim for oral stepdown therapy in gram-negative bacteremia: a multi-center, retrospective cohort study

**DOI:** 10.1017/ash.2026.10771

**Published:** 2026-06-29

**Authors:** Jordan Jones, Rachel B. Colven, Taylor Morrisette, Krutika Mediwala Hornback, Lindsay Deloney, Zachary Gruss, Gustavo Alvira-Arill, Tracie Delay, Aaron Hamby

**Affiliations:** 1 Pharmacy Services, MUSC Health: Medical University of South Carolina, USA; 2 Clinical Pharmacy & Outcomes Sciences, MUSC Health: Medical University of South Carolina, USA

## Abstract

**Objective:**

Many studies have evaluated the safety and efficacy of oral stepdown therapy for treatment of gram-negative bacteremia (GNB). There are currently no studies comparing the safety and effectiveness of various dosing strategies of sulfamethoxazole-trimethoprim (SMX-TMP) in these patients.

**Methods:**

This retrospective cohort study included adult patients at 6 hospitals within a health system with GNB, excluding *Stenotrophomonas spp.* that received at least 72 hours of oral SMX-TMP. Patients were grouped based on high- (≥8 mg/kg) or low-dose (<8 mg/kg) SMX-TMP. The primary outcome was a composite of all-cause mortality and recurrence at 30 days. Secondary outcomes included readmission, hyperkalemia requiring intervention, acute kidney injury, and intolerance leading to SMX-TMP discontinuation.

**Results:**

There were 176 patients included (25.6% high-dose, 74.4% low-dose) in this study. Baseline characteristics were similar except for age, sex, and dosing body weight. Median total duration of therapy for both groups was approximately 14 days; time to initiation of antibiotics was similar between groups. Six patients met the composite outcome (high-dose: 4.4% vs. low-dose: 3.1%; *P* = .646). Secondary outcomes did not differ significantly between groups.

**Conclusions:**

SMX-TMP is commonly used as oral stepdown therapy in GNB. Results of this study indicate that low-dose (<8 mg/kg) SMX-TMP may be sufficient, as outcomes were similar between the groups. To date, this is the first study evaluating different dosing strategies of SMX-TMP for this indication.

## Introduction

Bloodstream infections caused by gram-negative bacteria are becoming increasingly common in the United States and are associated with significant healthcare costs, morbidity, and mortality, with one study demonstrating a one-year all-cause mortality rate of 36.2%.^1,2^ Such infections are typically secondary processes, most commonly arising from a urinary tract or intra-abdominal source.^3^ Between 1997 and 2016, *Escherichia coli* and *Klebsiella pneumoniae* were the second- and third-most common pathogens recovered from all bloodstream infections in North America, following *Staphylococcus aureus*.^4^

Multiple studies have evaluated the safety and effectiveness of transitioning from intravenous (IV) to oral antibiotics in patients with gram-negative bacteremia (GNB), predominantly focusing on highly bioavailable beta-lactams, fluoroquinolones, and sulfamethoxazole-trimethoprim (SMX-TMP).^5–7^ Current guidance from the Infectious Diseases Society of America in partnership with the Society for Healthcare Epidemiology of America provides a strong recommendation for and highlights the potential benefits of transitioning patients from IV to oral therapy when clinically appropriate.^8^ The value of early IV to oral switch is supported by prior studies which have revealed no difference in outcomes for patients who received fewer than 5 days of IV therapy prior to definitive oral therapy for GNB, as well as significant reductions in line-related complications.^5,9^ Combined with society guideline recommendations, these data provide a strong platform for antimicrobial stewardship opportunities nationwide.

Although prior studies have demonstrated the effectiveness of SMX-TMP as oral stepdown therapy for GNB, little guidance exists for optimal dosing strategies, with few studies reporting the dosing regimens that patients received.^5–7^ Typical dosing recommendations vary greatly by indication but often feature dosing by tablet size (ie, 2 double-strength tablets twice daily; 640 mg/day TMP) or body weight (ie, 10 mg/kg/day TMP divided twice daily). Given the risk of accumulation and renal toxicity, SMX-TMP should be adjusted for renal function.^10–12^ Though no society guidance provides explicit dosing recommendations for SMX-TMP in patients with GNB, various institution-specific protocols are readily available which often recommend for 8–10 mg/kg/day TMP divided into two or three doses.^13,14^ Given the pharmacokinetic and pharmacodynamic properties of SMX-TMP, a higher daily dose (≥8 mg/kg/day TMP) may be needed to clinically cure patients with GNB; though to our knowledge, no studies have compared the safety and effectiveness of high- versus low-dose SMX-TMP for this indication.

The purpose of this study was to assess differences in effectiveness or safety outcomes associated with high-dose (≥ 8 mg/kg/day TMP) versus low-dose (<8 mg/kg/day TMP) SMX-TMP in patients with GNB. This study aims to add to current literature by evaluating different dosing strategies, as well as including patients who were immunocompromised, hemodynamically unstable, or those for whom source control was not achieved, as well as patients with polymicrobial GNB.^7,15,16^

## Methods

This was a multi-center, retrospective cohort study of adult patients (≥18 yr of age) admitted to the Medical University of South Carolina (MUSC) Health system, which includes a large academic tertiary referral center and nine regional campuses across South Carolina, between July 1, 2016, and June 31, 2024. Patients were included for review if they had documented GNB with isolated organisms that demonstrated in vitro susceptibility to SMX-TMP and received oral SMX-TMP for at least 72 hours. For polymicrobial GNB, all isolates must have demonstrated in vitro susceptibility to SMX-TMP. Patients were excluded if SMX-TMP was ordered for indications other than GNB, was used for infections caused by *Stenotrophomonas* species, and/or used in combination with other antibiotics that demonstrated in vitro activity against the infecting isolate, and for patients with documented hospice transfer or withdrawal of care within 72 hours of diagnosis of GNB. Because this research question has not been rigorously explored, there was no precedent established on which to base a *priori* power calculation; therefore, a power calculation was not performed.

Baseline and clinical characteristics, as well as microbiological culture information, were collected via manual chart review of individual electronic medical records (EMRs) and recorded in the Research Electronic Data Capture (REDCap) database.^17^ Data points included age, sex, ethnicity, allergies, height, weight, body mass index (BMI), severity of illness, treatment characteristics, laboratory values, and treatment outcomes. Severity of illness was determined by Pitt bacteremia scoring calculated at the time of index blood culture.^18^ Infections were categorized as complicated or uncomplicated. Complicated infections were defined by one or more of the following: unidentified source of infection, inability to obtain source control (if applicable), clinical instability at 72 hours despite appropriate antibiotic therapy, or occurrence in an immunosuppressed patient.^19^ Immunosuppressed patients were defined as those with any of the following: malignancy associated with chemotherapy administration within the past 28 days, neutropenia (absolute neutrophil count < 1,000 cells/mm^3^), history of solid organ transplantation, human immunodeficiency virus (HIV) with acquired immunodeficiency syndrome (AIDS), use of prednisone at a dose ≥ 20 mg daily for at least 14 days (or equivalent), or use of other immunosuppressive agents (eg, hydroxychloroquine, tacrolimus, mycophenolate mofetil). The presence of an acute kidney injury (AKI) was defined according to meeting at least stage 1 criteria of the 2012 Kidney Disease: Improving Global Outcomes (KDIGO) classification, AKI stage was not collected.^20^ Concomitant nephrotoxic agents were collected, which included but were not limited to vancomycin, angiotensin-converting enzyme (ACE) inhibitors, angiotensin II receptor blockers (ARBs), nonsteroidal anti-inflammatory drugs (NSAIDs), tacrolimus, and cyclosporine. Source control was defined as an intervention designed to remove the source of infection (ie, catheter removal, abscess drainage, lithotripsy, etc.); for infectious processes in which no formal definition for source control exists (ie, uncomplicated cystitis and pneumonia), the source control data point was not collected. The presence of an Infectious Diseases (ID) consultation was determined by formal documentation in the EMR. All sites had access to ID consultation services, including ID pharmacists, for the entirety of the study window.

In patients who received multiple active IV antibiotics prior to transitioning to oral SMX-TMP, all active agents were recorded. Data for mortality, recurrence, and readmission were all determined 30 days from the completion of SMX-TMP. Notably, due to the cascade reporting of antimicrobial susceptibilities at this institution, levofloxacin susceptibility was not known for all isolates. If an isolate demonstrated in vitro susceptibility to ciprofloxacin, susceptibility to levofloxacin was assumed.

Dosing for SMX-TMP was categorized as either high-dose (≥8 mg/kg/day TMP) or low-dose (<8 mg/kg/day TMP). Throughout the period of the study, institutional standardized dosing guidelines were not available. If a patient’s BMI was <30 kg/m^2^, their actual body weight was used to calculate their total daily dose; if their BMI was ≥30 kg/m^2^, the Devine formula was used to calculate their adjusted body weight, which was then used to calculate their total daily dose.^21,22^ The primary end point was a composite of 30-day all-cause mortality and 30-day recurrence of GNB with the presence of the same organism(s) species; secondary endpoints included 30-day all-cause readmission, AKI, hyperkalemia requiring intervention, and intolerance to SMX-TMP leading to discontinuation. Laboratory-associated secondary outcomes were only recorded for patients with follow-up labs documented. Normality of continuous data was assessed via the Shapiro-Wilk and Kolmogorov-Smirnov tests, skew, and kurtosis. To assess differences in the groups based on dosing, nominal data were compared with the chi-squared or Fisher’s exact test and continuous data were compared with the Mann-Whitney U or Student’s t-test. All statistical analyses were conducted via IBM SPSS software, version 28.0 (SPSS IBM Corp., Armonk, NY, USA). This study was reviewed and approved by the Institutional Review Board at MUSC Health via standard review prior to commencement.

## Results

**Figure 1.**
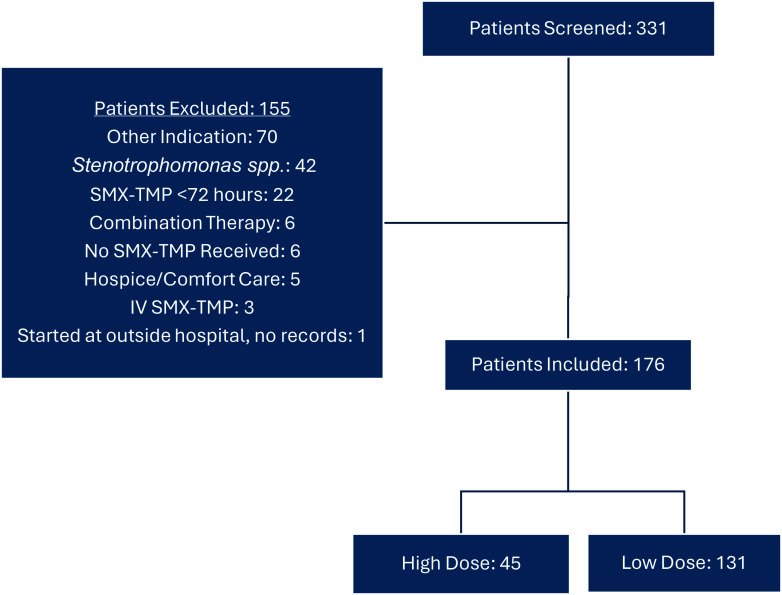
Screening and exclusion.

A total of 331 patients were assessed for inclusion, with 176 meeting criteria to be included. Of these, 45 patients received high-dose SMX-TMP and 131 patients received low-dose (Figure 1). Patient baseline characteristics are described in Table 1, with both groups being relatively well-balanced except for a greater proportion of female, younger patients and lower dosing body weight in the high-dose group. The groups did not differ significantly with regard to allergies, though five total patients reported an allergy to sulfa-containing medications prior to initiation of SMX-TMP; baseline sodium, potassium, and serum creatinine were similar and within normal ranges. Only three patients met predefined criteria for renal dose adjustment.

**Table 1.** Baseline characteristics Table 1 long description.

	High-dose (n = 45)	Low-dose (n = 131)	All (n = 176)	*P* value
Female sex, n (%)	28 (62.2)	55 (42.0)	83 (47.2)	.019
Age (years), median (IQR)	58.0 (39.0–69.0)	65.0 (56.0–75.5)	63.0 (50.8–74.0)	.014
Race/ethnicity, n (%)				.609
White	27 (60.0)	76 (58.0)	103 (58.5)	
African American	15 (33.3)	50 (38.2)	65 (36.9)	
Hispanic or Latino	3 (6.7)	4 (3.1)	7 (4.0)	
Other	0 (0.0)	1 (0.8)	1 (0.6)	
Dosing body weight (kg), median (IQR)	69.8 (59.0–75.8)	74.0 (65.5–83.7)	72.3 (63.7–81.7)	.008
Site, n (%)				.184
Location 1 (>700 beds)	44 (97.8)	105 (80.2)	149 (84.7)	
Location 2 (300–400 beds)	1 (2.2)	14 (10.7)	15 (8.5)	
Location 3 (300–400 beds)	0 (0.0)	5 (3.8)	5 (2.8)	
Location 4 (200–300 beds)	0 (0.0)	2 (1.5)	2 (1.1)	
Location 5 (100–200 beds)	0 (0.0)	1 (0.8)	1 (0.6)	
Location 6 (200–300 beds)	0 (0.0)	4 (3.1)	4 (2.3)	
Allergies, n (%)				
Penicillin	5 (11.1)	21 (16.0)	26 (14.8)	.422
Cephalosporin	0 (0.0)	1 (0.8)	1 (0.6)	>.999
Fluoroquinolone	1 (2.2)	8 (6.1)	9 (5.1)	.451
Sulfa	1 (2.2)	4 (3.1)	5 (2.8)	>.999
None of the above	38 (84.4)	101 (77.1)	139 (79.0)	.297
Source, n (%)				.157
Urinary tract	22 (48.9)	85 (64.9)	107 (60.8)	
Intra-abdominal	12 (26.7)	32 (24.4)	44 (25.0)	
Line-related	3 (6.7)	4 (3.1)	7 (4.0)	
Respiratory	1 (2.2)	1 (0.8)	2 (1.1)	
Unknown	5 (11.1)	5 (3.8)	10 (5.7)	
Other	2 (4.4)	4 (3.1)	6 (3.4)	
Pitt bacteremia score, median (IQR)	1 (0–2)	2 (1–3)	1 (0–3)	.051
Baseline potassium (mEq/L), median (IQR)	3.8 (3.6–4.1)	3.8 (3.5–4.1)	3.8 (3.5–4.1)	.673
Baseline serum creatinine (mg/dL), median (IQR)	0.8 (0.6–1.0)	0.9 (0.7–1.1)	0.8 (0.7–1.1)	.090
Renal dose adjustment needed for creatinine clearance <30 mL/min or hemodialysis	1 (0.02)	2 (0.02)	3 (0.02)	>.999
Concomitant nephrotoxic agent(s), n (%)	14 (31.1)	49 (37.4)	63 (35.8)	.447
Complicated BSI, n (%)	13 (28.9)	39 (29.8)	52 (29.6)	.911
Unidentified Source	4 (8.9)	4 (3.1)	8 (17.4)	.206
Inability to obtain source control	5 (11.1)	10 (7.6)	15 (32.6)	.537
Clinical instability at 72 hours	1 (2.2)	1 (0.8)	2 (4.4)	.447
Immunocompromised	5 (11.1)	25 (19.1)	30 (17.1)	.220
Malignancy	1 (2.2)	16 (12.2)	17 (9.7)	.075
Transplant	3 (6.7)	5 (3.8)	8 (4.6)	.423
Steroids or other meds	1 (2.2)	3 (2.3)	4 (2.3)	>.999
HIV/AIDS	0 (0.0)	2 (1.5)	2 (1.1)	>.999
Neutropenia	0 (0.0)	0 (0.0)	0 (0.0)	–
Transplant	3 (6.7)	5(3.8)	8 (4.6)	>.999
Kidney	3 (6.7)	4 (3.1)	7 (4.0)	.374
Liver	0 (0.0)	1 (0.8)	1 (0.6)	>.999

AIDS, acquired immunodeficiency syndrome; BSI, bloodstream infection; HIV, human immunodeficiency virus; IQR, interquartile range.

The most common sources of infection in both groups were of urinary tract and intra-abdominal origin, with numerically more patients in the low-dose group (64.9%) having a urinary source than in the high-dose group (48.9%) and numerically more patients in the high-dose group having an unknown source (11.1%) than in the low-dose group (3.8%). For patients with an applicable source (n = 99), source control was achieved in 67 (67.7%) and differed significantly between groups, being more common in the low-dose group (52/70, 74.3%) than the high-dose group (15/29, 51.7%) (*P* = .029). Patients in the low-dose group also demonstrated a numerically higher severity of illness based on median Pitt bacteremia score (median 2, IQR 1–3), compared with the high-dose group (median 1, IQR 0–2) (*P* = .051). The most commonly isolated organism was *E. coli*, followed by *K. pneumoniae*, and *Proteus mirabilis* (Table 2). The distribution of organisms and proportion of those with polymicrobial infections did not differ significantly between the two dosing groups. Overall susceptibility patterns were similar between the groups and are described in Table S1. There was an overall trend of decreased susceptibility to beta-lactams in this cohort when compared with fluoroquinolones and SMX-TMP.

**Table 2.** Organism information

	High-dose (n = 45)	Low-dose (n = 131)	All (n = 176)	*P* value
Organism, n (%)				
* Escherichia coli*	22 (48.9)	76 (58.0)	98 (55.7)	.288
*Klebsiella pneumoniae*	10 (22.2)	35 (26.7)	45 (25.6)	.551
*Proteus mirabilis*	1 (2.2)	10 (7.6)	11 (6.3)	.294
*Enterobacter cloacae*	3 (6.7)	1 (0.8)	4 (2.3)	.052
*Klebsiella oxytoca*	1 (2.2)	2 (1.5)	3 (1.7)	>.999
Other	9 (20.0)	14 (10.7)	23 (13.1)	.110
Polymicrobial	1 (2.2)	6 (4.6)	7 (4.0)	.680

The treatment characteristics for both groups were similar and are outlined in Table 3. The median (IQR) dose of SMX-TMP was 9.4 (8.8–10.7) mg/kg/day TMP in the high-dose group and 4.6 (4.0–5.3) mg/kg/day TMP in the low-dose group (*P* < .001). There were no significant differences between groups with regards to duration or time to initiation of antibiotics. The choice of initial IV therapy was mostly similar between the groups, with most patients receiving ceftriaxone and/or piperacillin-tazobactam. Some patients were escalated or de-escalated to other IV agents prior to oral stepdown therapy with SMX-TMP. The only significant difference between the groups regarding initial IV therapy was the increased use of meropenem in the high-dose group (17, 37.8%) compared with the low-dose group (13, 9.9%) (*P* < .001). Additionally, more patients in the high-dose group (24, 53.5%) received formal ID consultation than in the low-dose group (43, 32.8%) (*P* = .015).

**Table 3.** Treatment characteristics

	High-dose (n = 45)	Low-dose (n = 131)	All (n = 176)	*P* value
Initial active IV antibiotics, n (%)				
Ceftriaxone	27 (60.0)	80 (61.1)	107 (60.8)	.899
Piperacillin-tazobactam	28 (62.2)	75 (57.3)	103 (58.8)	.559
Cefepime	6 (13.3)	33 (25.2)	39 (22.2)	.098
Meropenem	17 (37.8)	13 (9.9)	30 (17.1)	<.001
Ertapenem	0 (0.0)	1 (0.8)	1 (0.6)	>.999
Other	4 (8.9)	17 (13.0)	21 (11.9)	.465
Time to antibiotics (hours), median (IQR)	1.3 (0.2–7.7)	1.1 (0.3–3.7)	1.1 (0.3–4.7)	.657
Duration of IV therapy (days), median (IQR)	2.8 (2.1–4.3)	3.3 (2.3–4.5)	3.1 (2.2–4.5)	.265
Duration of therapy with SMX-TMP (days), median (IQR)	10.5 (7.6–13.5)	9.1 (6.6–12.3)	10.0 (7.1–12.5)	.216
Duration of antibiotic therapy (days), median (IQR)	14.1 (12.1–16.0)	14.3 (11.0–16.5)	14.2 (11.3–16.3)	.845
Total daily dose TMP in mg/kg/day, median (IQR)	9.4 (8.8–10.7)	4.6 (4.0–5.3)	4.9 (4.2–8.1)	<.001
Hospital length of stay (days), median (IQR)	5 (4–8)	5 (3–8)	5 (3.8–8)	.516
ICU admission, n (%)	10 (22.2)	32 (24.4)	42 (23.9)	.765
ICU length of stay (days), median (IQR)	2.5 (2–5)	4 (3–6)	4 (2.3–6)	.267
Follow-up labs drawn, n (%)	18 (40.0)	48 (36.6)	66 (37.5)	.688
Infectious diseases consultation, n (%)	24 (53.3)	43 (32.8)	67 (38.1)	.015
Source control achieved, n/N (%)	15/29 (51.7)	52/70 (74.3)	67/99 (67.7)	.029

ICU, intensive care unit; IV, intravenous; IQR, interquartile range; SMX-TMP, sulfamethoxazole-trimethoprim.

Six patients in total met the composite outcome, as shown in Table 4, with one death and one recurrence in the high-dose group and four deaths in the low-dose group (*P* = .645). Though more deaths occurred in the low-dose group, only one was related to an infectious cause (Table S2). No significant differences were found between groups with the primary or secondary outcomes assessed.

**Table 4.** Outcomes

	High-dose (n = 45)	Low-dose (n = 131)	All (n = 176)	*P* value
Primary outcomes, n (%)				
Composite	2 (4.4)	4 (3.1)	6 (3.4)	.646
Mortality	1 (2.2)	4 (3.1)	5 (2.8)	>.999
Recurrence	1 (2.2)	0 (0.0)	1 (0.6)	.256
Secondary outcomes, n/N (%)				
Readmission^a^	11/43 (25.6)	38/130 (29.2)	49/173 (28.3)	.645
Acute kidney injury^b^	3/18 (16.7)	9/48 (18.8)	12/66 (18.2)	>.999
Hyperkalemia requiring intervention^b^	0/18 (0.0)	2/48 (4.2)	2/66 (3.0)	>.999
Any intolerance	2 (4.4)	3 (2.3)	5 (2.8)	.603
SMX-TMP discontinued	3 (6.7)	2 (1.6)	5 (2.7)	.603

SMX-TMP, sulfamethoxazole-trimethoprim.

a Three patients (two high-dose, one low-dose) were still admitted at 30 days.

b Denominator based on number of patients who received follow-up labs.

Overall, 49 patients were readmitted within 30 days of their index admission. Reasons for readmission varied, the majority of which were not related to their initial infectious process.

## Discussion

To our knowledge, this is the first study to evaluate the safety and effectiveness of different dosing strategies for SMX-TMP in patients with GNB. Based on the results of this cohort, there seem to be no clinically significant differences between patients who received high- versus low-dose SMX-TMP as oral stepdown therapy for the treatment of GNB.

Prior studies have established the role of SMX-TMP for this indication, but few have dosing data available.^6,7^ One study evaluating various agents for oral stepdown therapy in GNB reported that all patients who received SMX-TMP were prescribed 640 mg TMP per day, but information on the patients’ weights was not reported.^5^ Typically, standard adult antibiotic dosing is based on a historical weight assumption of 70kg; if we apply this assumption to a standardized regimen of 640 mg TMP daily, the total daily dose of TMP would be 9.1 mg/kg/day.^23^ Based on the results of this study, dosing of SMX-TMP in patients with GNB may not need to be this aggressive, as evidenced by patients in the low-dose group receiving approximately half of this dose without experiencing worse outcomes. Notably, patients in the low-dose group tended to have a higher dosing body weight, which is not unexpected.

There is some clinical controversy surrounding the most appropriate weight-based dosing strategy for SMX-TMP, specifically with regard to dosing in patients with obesity, as drug exposure changes with body size.^24^ As there is a paucity of clinical outcomes data available, most of these decisions are guided by pharmacokinetic and pharmacodynamic properties and safety concerns of SMX-TMP, thus introducing the possibility of selection bias on the prescribers’ behalf.^22^ To mitigate the risk of adverse effects with high doses, especially hyperkalemia, we evaluated adjusted body weight for dosing in patients whose BMI is ≥30 kg/m^2^. Because so few patients met the primary outcome of the study, it is difficult to determine whether the use of adjusted body weight played a role in the outcomes of this study.

With relatively few oral options available for GNB, especially in this cohort which demonstrated overall lower susceptibility to beta-lactams, safety and tolerability become a significant concern when choosing an agent. Though usually well tolerated, oral beta-lactams may perform worse than SMX-TMP or fluoroquinolones for this indication because of their relative lower bioavailability.^5^ Fluoroquinolones, although highly bioavailable, possess significant adverse effects and drug-drug interactions.^25,26^ Additionally, adverse effects from fluoroquinolones may be more prevalent in elderly patients, which makes their use less appealing in that population.^27,28^ Likewise, as renal function declines with age, it may be postulated that adverse effects associated with SMX-TMP may be more prevalent in elderly patients.^29,30^ However, despite the median age of patients in this cohort, SMX-TMP was well tolerated in both groups, although high dosing was typically avoided in older patients. A prior study demonstrated that electrolyte disorders occurred more commonly in patients receiving higher doses and those with renal dysfunction. Few patients in this cohort received follow-up labs, so the true prevalence of electrolyte abnormalities and AKI may be underreported; however, this reflects real-world practice as patients may be transitioned to oral therapy at discharge without follow-up. Surprisingly, there were few patients in both groups who experienced AKI despite 35.8% being on concomitant nephrotoxic agents, predominantly NSAIDs and ACE inhibitors or ARBs.

Importantly, there were treatment characteristics that were not balanced between the groups. Notably, more patients who received an ID consultation also received high-dose SMX-TMP, likely mediated by formal dosing recommendations from ID-trained pharmacists. Studies have shown a significant mortality benefit in patients with GNB who receive an ID consultation, whereas prior data were only available for gram-positive bacteremia and candidemia.^31,32^ The use of empiric meropenem was also more common in the high-dose group, for which no identifiable explanation could be found. According to the Pitt bacteremia scores, the severity of illness in the high-dose group was actually lower than patients in the low-dose group, though not statistically significant. Source control was achieved less often in patients who received high-dose SMX-TMP, and overall, only achieved by roughly two-thirds of patients in this cohort. However, current guidance from the Infectious Diseases Society of America indicates that for patients with complicated urinary tract infections, treatment can mimic that of uncomplicated cystitis once source control is achieved, with the duration being set based on the day of source control.^33^ Based on this recommendation, it may have been reasonable to consider uncomplicated cystitis and having source control on day one of appropriate therapy; however, this precedent has not yet been established in any studies to date. In clinical practice, these patients are not treated as having complicated GNB; thus, we did not consider them to have a lack of source control. Of note, the low-dose group had more patients with a urinary source and fewer patients with an unknown source, which may have introduced selection bias on the prescriber’s behalf as previously described.

Treatment characteristics were overall similar between the groups, though patients in the low-dose group exhibited a numerically higher severity of illness. The median time to initiation of antibiotics was aligned with the Surviving Sepsis Campaign recommendations, and patients were transitioned to oral antibiotics rather quickly.^34^ Comprehensive data with prospective studies regarding shorter durations of therapy for GNB were not common until recently, so implementation of these results had not yet been adopted into practice at the time of treatment for many patients in this cohort.^35–37^ Though few patients experienced intolerance to SMX-TMP, it is unclear if a re-evaluation would yield the same primary outcomes in a cohort whose course was more aligned with a seven-day duration.

This study adds to current literature by evaluating different dosing strategies and including patients with polymicrobial GNB, immunocompromised patients, and critically ill patients. In addition to being multicenter and including community hospitals, this study is also strengthened by having more patients in the low-dose group. Additionally, having few exclusion criteria strengthens the real-world impact and will allow the results of this study to be generalizable to various patient populations. This study is limited by its small sample size, retrospective nature, and inability to identify patients who presented to outside hospitals for a subsequent admission; outpatient adherence following hospitalization was also difficult to assess. Furthermore, a power calculation was not performed, so it is possible that our study may be underpowered, and 95% confidence intervals were not reported to show the degree of imprecision around the estimate. Additionally, differences between dosing groups were not adjusted for because of the small number of outcome events; therefore, the findings should be interpreted as exploratory or descriptive, rather than definitive evidence of comparative effectiveness or safety. Overall, the results of this retrospective cohort analysis suggest that a lower total daily dose of SMX-TMP may be effective as definitive oral therapy for patients with GNB, especially in those with documented source control. Future studies are needed to assess treatment outcomes with low-dose SMX-TMP for the treatment of GNB in patients who receive less than 14 days of therapy.

The results of this study suggest that patients with GNB may be successfully treated with less than 8 mg/kg/day TMP. Notably, these results are primarily from patients with GNB secondary to UTIs and intra-abdominal infections, and in patients for whom treatment duration was 14 days. Further, more robust studies with larger cohorts are needed to determine if these results would be confirmed with shorter durations of therapy, which are now more common.

## Supporting information

10.1017/ash.2026.10771.sm001 Jones et al. supplementary material Jones et al. supplementary material

## Supplementary material

The supplementary material for this article can be found at https://doi.org/10.1017/ash.2026.10771.

## Research transparency and reproducibility

The data set is available from the corresponding author upon reasonable request.

## Table 1 Long description

A table comparing baseline characteristics of patients receiving high-dose and low-dose SMX-TMP. The table has 25 rows and 5 columns. Column headers are High-dose (n = 45), Low-dose (n = 131), All (n = 176), and P value. Row labels include Female sex, Age, Race/ethnicity, Dosing body weight, Site, Allergies, Source, Pitt bacteremia score, Baseline potassium, Baseline serum creatinine, Renal dose adjustment needed, Concomitant nephrotoxic agent(s), Complicated BSI, Unidentified Source, Inability to obtain source control, Clinical instability at 72 hours, Immunocompromised, Malignancy, Transplant, Steroids or other meds, HIV/AIDS, Neutropenia, Transplant, Kidney, and Liver. Each row provides specific data points for each category. Notable trends include a higher proportion of female and younger patients in the high-dose group and a lower dosing body weight. The groups are relatively well-balanced in terms of allergies, baseline sodium, potassium, and serum creatinine levels.

Navigate back to Table 1.
