# Supplementary material for: Assessment of treatment outcomes in patients receiving high- versus low-dose sulfamethoxazole-trimethoprim for oral stepdown therapy in gram-negative bacteremia: a multi-center, retrospective cohort study
Source: Antimicrob Steward Healthc Epidemiol. 2026 Jun 29;6(1):e195. doi: 10.1017/ash.2026.10771 (PMC13312236; doi:10.1017/ash.2026.10771)
Supplement: Jones et al. supplementary material [file S2732494X26107712sup001.docx]

Supplemental Table 1. Susceptibilities

|  | High-Dose (n=45) | Low-Dose (n=131) | All (n=176) | P Value |
| --- | --- | --- | --- | --- |
| Ampicillin, n (%) | 16 (35.6) | 49 (37.4) | 65 (36.9) | 0.825 |
| Ampicillin-Sulbactam, n (%) | 21 (46.7) | 81 (61.8) | 102 (58.0) | 0.075 |
| Cefazolin, n (%) | 24 (53.3) | 81 (61.8) | 105 (59.7) | 0.316 |
| Ciprofloxacin, n (%) | 30 (66.7) | 89 (67.9) | 119 (67.6) | 0.875 |
| Levofloxacin, n/N (%) | 29/33 (87.9) | 90/98 (91.8) | 119/131 (90.8) | 0.829 |

Supplemental Table 2. All-Cause Mortality

| Age | Sex | Group | Initial GNB Causative Organism | Cause of Mortality |
| --- | --- | --- | --- | --- |
| 75 | M | High | *Escherichia coli* | Progression of malignancy (cholangiocarcinoma) |
| 63 | F | Low | *Acinetobacter baumannii* | Septic shock, VRE and *E. coli* (SMX-TMP resistant) bacteremia secondary to GI bleed |
| 65 | F | Low | *Klebsiella aerogenes* | Progression of malignancy (metastatic uterine cancer) |
| 72 | M | Low | *Escherichia coli* | Progression of malignancy (metastatic pancreatic cancer) |
| 87 | M | Low | *Klebsiella pneumoniae* | Aspiration event during procedure, code, AHRF, care withdrawn |
| AHRF: acute hypoxic respiratory failure  GI: gastrointestinal  GNB: Gram-negative bacteremia  SMX-TMP: sulfamethoxazole-trimethoprim  VRE: vancomycin-resistant *Enterococcus faecium* | | | | |
